# Maternal nucleotide supplementation improves the intestinal morphology and immune function in lipopolysaccharide-challenged newborn piglets

**DOI:** 10.3389/fvets.2022.1043842

**Published:** 2022-10-26

**Authors:** Qiming Li, Ifen Hung, Kaiwen Bai, Tian Wang

**Affiliations:** ^1^College of Animal Science and Technology, Nanjing Agricultural University, Nanjing, China; ^2^Anyou Biotechnology Group Co., Ltd., Suzhou, China

**Keywords:** late gestation sow, newborn piglet, nucleotides, lipopolysaccharide, intestinal morphology, intestinal inflammatory response

## Abstract

This study aimed to evaluate the effects of maternal nucleotide (NT) supplementation on intestinal morphology and immune function in lipopolysaccharide-challenged newborn piglets. At 85 d gestation, 12 sows were selected and assigned to two groups: the CON group (basal diet, *n* = 6) and the NT group (basal diet with 1 g/kg NT mixture, *n* = 6). After parturition, newborn piglets were collected without suckling. Piglets from the CON group were intraperitoneally injected with sterile saline or lipopolysaccharide (LPS, 10 mg/kg body weight), and divided into the C-CON (*n* = 6) and C-LPS groups (*n* = 6). Piglets from the NT group received the same treatment and were divided into the N-CON (*n* = 6) and N-LPS groups (*n* = 6). The blood and small intestinal samples of piglets were collected 1 h after injection. The results showed that: (1) maternal NT supplementation increased the concentrations of serum complement C3 and C4 (*P* < 0.05), and suppressed the increase in serum hypersensitive C-reactive protein in LPS-challenged newborn piglets (*P* < 0.05); (2) maternal NT supplementation increased the villus height and the ratio of villus height to crypt depth in the duodenum of newborn piglets (*P* < 0.05) and inhibited the LPS-induced decrease in the villus height in the jejunum and ileum (*P* < 0.05). (3) The LPS-induced increased levels of interleukin-6 in the jejunum and tumor necrosis factor-α in the ileum of newborn piglets were suppressed by maternal NT supplementation (*P* < 0.05). (4) In the jejunum of newborn piglets, maternal NT supplementation inhibited the LPS-induced increase in toll-like receptor 4 (TLR4) mRNA and protein expression (*P* < 0.05) and the decrease of nuclear factor-κB inhibitor α (IκBα) protein expression (*P* < 0.05). In the ileum, piglets had a lower nuclear factor-κB (NFκB) mRNA expression in the NT groups than the CON groups (*P* < 0.05), and maternal NT supplementation suppressed the decrease of IκBα mRNA in LPS-treated piglets (*P* < 0.05). In conclusion, maternal NT supplementation could promote the intestinal development and immune function of newborn piglets, and may improve LPS-induced intestinal inflammatory responses *via* the TLR4/IκBα/NFκB pathway.

## Introduction

Nucleotides (NTs) are intracellular compounds involved in almost all biological processes in the body (1). Through two endogenous synthetic pathways, including the *de novo* pathway and the salvage pathway, NTs are produced to meet the needs of healthy individuals, which is why NTs are called non-essential components (2). However, intestinal epithelial cells, lymphoid cells, and other cells that turn over rapidly lack the ability to produce NTs *via* the *de novo* pathway (3). And under certain conditions, such as periods of rapid growth, immunosuppression, or limited nutrient intake, the body has increased demands for NTs (4). Consequently, it is essential to obtain exogenous NTs to compensate for the shortage of endogenous NTs in some cases.

In the modern swine industry, piglets grow rapidly and are always stimulated by various stresses during the growth period, such as vaccination, weaning, and transportation. And after weaning, piglets no longer receive sow milk rich in NTs, thus losing an important source of NTs. Simultaneously, few NTs are present in the basic feed ingredients (5). This practical problem results in a lack of NT supply to piglets. Therefore, many studies have investigated the feasibility of using exogenous NTs in basic piglet diets. Results have shown that dietary NTs could improve growth performance (6, 7) and decrease the diarrhea rate in weaned piglets (8). From embryo formation to weaning, sows play an important role in piglet development and provide nutrition to fetal pigs through the placenta. Some reports have shown that adding NTs to the gestation diets of sows could increase the NT content in sow milk (9) and the number of weaned piglets per litter (10); however, few studies have investigated the effects of maternal NT supplementation on the development of newborn piglets. In addition, after sows' delivery, newborn piglets leave the intrauterine environment and enter a colder, drier, and microbially richer extrauterine environment. Changes in the environment may lead to a deterioration in the health of piglets and put them at a disadvantage in competition with other individuals (11). The intestine has a large area of contacting with the external environment and can be directly affected by environmental changes. As an important organ in piglets' bodies, intestinal integrity is the fundamental for the digestion and absorption of nutrients, and the intestinal barrier function and immune functions protect the healthy development of newborn piglets. Previous studies have demonstrated that dietary NT supplementation could promote the development of the intestinal and immune systems of weaned piglets (12, 13). However, whether maternal NT supplementation affects the intestinal morphology and immune function of newborn piglets requires further study.

Lipopolysaccharide (LPS) is a major component of the outer membrane of Gram-negative bacteria. Appropriate concentrations of LPS can regulate the immune system; however, excess LPS in the body can trigger a severe inflammatory response and lead to tissue and vascular damage (14). Currently, excess of LPS is widely used to construct experimental animal models to simulate microbial infection or immunological stress (15). In this experiment, we fed the late gestation sows with the basal diets or the basal diets that contained exogenous NTs until sows' parturition, obtained newborn piglets that had not received colostrum and stimulated them with LPS. Our study aimed to evaluate the effects of maternal NT supplementation on intestinal morphology and immune function in lipopolysaccharide-challenged newborn piglets and to provide novel insights into the application of maternal exogenous NTs supplementation in the swine industry.

## Materials and methods

### Animals, diets, and experimental design

This work was conducted in Shanxian Heyou Ruian Agricultural Development Co., Ltd. (Sanmenxia, China). Twelve [Duroc × (Landrace × Yorkshire)] sows with similar parity and farrowing time were selected at 85 d of gestation. Six sows were allocated to the CON group (fed a basal gestational diet), and the other six were assigned to the NT group (fed a basal gestational diet supplemented with 1.0 g NT mixture per kg of diet). All the sows were housed individually in 2.1 × 0.7 m floor pens with ambient temperature controlled at 25°C. From 85 d of gestation to the farrowing day, sows were fed twice a day (07:00 and 14:00) with a total of 3.5 kg/d gestational diets and free access to water. The gestation diet based on the National Research Council (16) guidelines was formulated to meet the nutrient requirements of the gestational sows (Table 1). The NTs used in this study were provided by Nanjing Biotogether CO., Ltd. (Nanjing, China) and consisted of adenosine 5′-monophosphate (5′AMP), uridine 5′-monophosphate (5′UMP), guanosine 5′-monophosphate disodium salt (5′GMP-Na_2_), cytidine 5′-monophosphate disodium salt (5′CMP-Na_2_), and inosine 5′-monophosphate disodium salt (5′IMP-Na_2_). The NT mixture added to the basal diet was prepared by mixing five mononucleotides in equal proportions (1: 1: 1: 1: 1).

**Table 1:** Ingredients and composition of gestation diets (as-fed basis).

**Items**	**Composition, %**
**Ingredients**	
Corn	51.40
Soybean meal	18.40
Bran	7.50
Rice bran meal	4.00
Wheat flour	10.00
Fish meal	1.50
Soy phospholipid flour	1.00
Soybean oil	2.20
Premix^1^	4.00
**Nutrition level** ^**2,3**^	
ME, MJ/kg	13.40
NE, MJ/kg	10.13
Crude protein	16.50
Crude fiber	3.50
Ca, %	0.85
Total P, %	0.60

^1^Premix supplied the following per kg complete diet: vitamin A, 8 000 IU; vitamin D3, 2 000 IU; vitamin E, 40 mg; vitamin K, 2 mg; vitamin B1, 3 mg IU; vitamin B2, 5 mg; vitamin B6, 3 mg; vitamin B12, 0.04 mg; nicotinic acid, 30 mg; vitamin B5, 20 mg; folic acid, 1.5 mg; biotin 0.3 mg; riboflavin 8 mg; Fe, 100 mg; Cu, 20 mg; Zn, 80 mg; Mn, 40 mg; I, 0.3 mg; Se, 0.25 mg.

^2^ME, metablizable energy; NE, net energy.

^3^Calculated value.

After parturition, the birth weight and the number of newborn piglets in each litter were immediately recorded. Subsequently, two male piglets with medium body weight in one litter of the CON group were selected and allocated to the C-CON group and C-LPS group, and two male piglets with mid-body weight in one litter of the NT group were selected and allocated to the N-CON group and N-LPS group. Finally, twenty-four newborn piglets were divided into four groups (*n* = 6). After that, piglets in the C-LPS group and N-LPS groups were intraperitoneally injected with 10 mg/kg LPS (17), and piglets in the C-CON group or N-CON group were injected with the same volume of sterile saline. LPS used in our study was obtained from Sigma-Aldrich Chemical Co. (L2880; St. Louis, MO, USA). Blood samples were collected from each piglet through the jugular vein into the vacutainer 1 h after injection with LPS or sterile saline. Following this procedure, the piglets were stunned by electric shock and slaughtered by exsanguination. Then, the small intestine was removed from the abdominal cavity of each piglet, and before collecting the duodenum (5 cm behind the pylorus), jejunum (5 cm before the end of jejunum Peyer's patches), and ileum (5 cm from the ileocecal valve) samples, the intestinal content was removed with saline. The samples (2 cm) taken from each segment of small intestine were put into 4% paraformaldehyde for histological analysis, and the remainder of the jejunal and ileal segments were immediately frozen in liquid nitrogen and preserved at −80°C for cytokine level, gene expression, and protein expression analyses.

### Serum parameters analysis

The serum samples were separated from blood by centrifugation at 3,500 × g for 10 min at 4°C. After that, commercial kits were used to detect serum alkaline phosphatase (ALP), alanine aminotransferase (ALT), and aspartate aminotransferase (AST) levels according to the manufacturer's instructions (Nanjing Jiancheng Bioengineering Institute, Nanjing, China). The serum concentrations of hypersensitive C-reactive protein (hs-CRP), complement C3 (C3), complement C4 (C4), cyclooxygenase 2 (COX-2), tumor necrosis factor α (TNF-α), interleukin 1β (IL-1β), interleukin 6 (IL-6), and interleukin 10 (IL-10) in the serum were detected using porcine-specific ELISA kits (Ruixin, Quanzhou, China).

### Intestinal morphology analysis

After fixation for 24 h in 4% paraformaldehyde, intestinal samples were dehydrated with a graded series of ethanol and cleared by xylene, then the samples were embedded in paraffin. Each sample was sliced into 5 μm thick sections and stained with hematoxylin and eosin. Ten well-oriented villi per section were selected using a Nikon ECLIPSE 80i light microscope (Tokyo, Japan). Villus height (VH) and crypt depth (CD) were measured by Image-Pro Plus software (Media Cybernetics, Inc., Rockville, MD, USA), and the ratio of villus height to crypt depth was defined as VCR (18).

### Goblet cell density analysis

The sections used in Alcian Blue/periodic acid Schiff stain (AB-PAS) were prepared in the same way as for the intestinal morphology analysis. The deparaffinized and rehydrated sections were stained with Alcian Blue solution for 5 min, gently washed in double-distilled H_2_O for 2 min, oxidized with the periodic acid solution for 15 min, and rinsed twice in double-distilled H_2_O. The sections were then placed in a Schiff solution for 30 min and rinsed with running water for 5 min. After hematoxylin staining and dehydration, the sections were sealed with neutral gum. The intestinal images were obtained using the same light microscope as previously described, and ten well-oriented villi per section were selected to calculate the goblet cell density. The final results were expressed as goblet cell (GC) number per 100 μm of villus length (19).

### Intestinal cytokine levels analysis

To measure intestinal cytokine levels, the tissue homogenates were prepared by homogenizing the frozen intestinal tissues with pre-cooled sterile saline at a ratio of 1:4 (wt/vol). Then, the homogenates were centrifuged at 3,500 × g for 10 min at 4°C. The supernatants were separated and diluted with sterile saline to obtain the optimal content. The supernatants' protein concentration was determined using a bicinchoninic acid (BCA) protein assay kit (Beyotime, Shanghai, China), and the concentrations of TNF-α, IL-1β, and IL-6 in the jejunum and ileum were detected using the corresponding ELISA kits, following the manufacturer's instructions (Ruixin, Quanzhou, China).

### RNA extraction and real-time quantitative PCR analysis

Total RNA was isolated from the jejunum and ileum with Total RNA Extraction Reagent (Vazyme Biotech, Nanjing, China). After measuring the purity and concentration of RNA using a spectrophotometer (NanoDrop Products, Wilmington, DE, USA), the final RNA concentration was adjusted to 500 ng/uL, and 1 μg of RNA was used for reverse transcription to obtain cDNA with a HiScript III RT SuperMix (Vazyme Biotech, Nanjing, China) for RT-qPCR according to the manufacturer's instructions. Then, cDNA was used to analyze the gene transcript abundance using quantitative real-time PCR with ChamQ SYBR qPCR master mix reagents (Vazyme Biotech, Nanjing, China). The sequences of primers are listed in Table 2, and GAPDH was used as the reference gene. The RT-qPCR data were analyzed using the 2^−ΔΔCt^ method (20).

**Table 2:** Primer sequences used in real-time quantitative PCR.

**Name^1^**	**Accession No**.	**Sequence (5′to 3′)**	**Size (bp)**
TLR4	NM_001113039.2	F: TCCCTGACAACATCCCCACATC	191
		R: TCCCGTCAGTATCAAGGTGGA	
NFκB	NM_001114281.1	F: TCAGCGCATCCAGACCAACA	87
		R: CAGAGCCGCACAGCATTCAG	
IκBα	NM_001005150.1	F: CAGGGCTACTCCCCGTACCA	180
		R: TCCAAGCACGCAGTCGTCATA	
IL-1β	NM_214029.1	F: GCCAGCTATGAGCCACTTCC	128
		R: TGACGGGTCTCGAATGATGCT	
IL-6	NM_012589.2	F: CCCAACTTCCAATGCTCTCCT	71
		R: GGATGGTCTTGGTCCTTAGCC	
TNF-α	NM_214022.1	F: ATCGGCCCCCAGAAGGAAGAG	351
		R: GATGGCAGAGAGGAGGTTGAC	
GAPDH	NM_001206359.1	F: CCAAGGAGTAAGAGCCCCTG	94
		R: AAGTCAGGAGATGCTCGGTG	

^1^TLR4, toll-like receptor 4; NFκB, nuclear factor kappa B; IκBα, nuclear factor kappa B inhibitor alpha; IL-1β, interleukin 1 beta; IL-6, interleukin 6; TNF-α, tumor necrosis factor alpha; GAPDH, glyceraldehyde-3-phosphate dehydrogenase.

### Western blotting analysis

The jejunal and ileal samples were treated with radioimmunoprecipitation assay (RIPA) lysis buffer (Beyotime, Shanghai, China), and the protein concentration in the intestinal tissue was determined using a BCA protein assay kit (Beyotime, Shanghai, China). Twenty micrograms of protein were loaded in each lane and separated on 10% sodium dodecyl sulfate polyacrylamide gel electrophoresis (SDS-PAGE) gels (Epizyme, Shanghai, China). After electrophoresis, the proteins were transferred from the target gel to the polyvinylidene fluoride (PVDF) membrane. Then, the PVDF membranes were blocked in 5% skim milk for 1.5 h, followed by incubation with primary antibodies against TLR4 (1:1000, 19811-1-AP, Proteintech Group, Wuhan, China), NF-κB (1:1000, 10745-1-AP, Proteintech), IκB-α (1:1000, 10268-1-AP, Proteintech), and GAPDH (1:7000, 10494-1-AP, Proteintech) overnight at 4°C. The membranes were then washed five times with Tris-buffered saline (TBS) containing 0.1% Tween 20 (TBST) buffer and incubated with the corresponding secondary antibody at 25°C for 1.5 h. After another five washes, the target protein expression was detected using ChemiDoc MP Imaging System (Bio-Rad Laboratories, Hercules, CA, USA) with ultrasensitive chemiluminescence (ECL) reagent (Biosharp, Hefei, China).

### Statistical analysis

SPSS 25.0 (IBM SPSS Company, Chicago, IL, USA) was used for all statistical analyses. The data were analyzed by ANOVA using a 2 × 2 factorial arrangement of treatments with the GLM procedure. The statistical model included the effects of maternal diet (Diet), LPS challenge (LPS), and their interactions (Diet × LPS). When the interaction was significant, the data were reanalyzed by one-way analysis of variance and Duncan's *post-hoc* test. Statistical differences were determined at *P* < 0.05, and a trend was considered at 0.05 < *P* < 0.10. The measurements were expressed as means with their standard errors unless otherwise stated.

## Results

### Serum parameters analysis

As shown in Table 3, piglets treated with LPS had lower C3 levels (*P* < 0.05) and higher serum ALT values (*P* < 0.05) than piglets injected with sterile saline. Maternal NT supplementation increased the serum C3 and C4 levels in newborn piglets (*P* < 0.05). In addition, there was an interaction effect observed between the maternal diet and LPS challenge on hs-CRP content (*P* < 0.05) and ALP value (*P* < 0.05). Serum hs-CRP levels were elevated (*P* < 0.05) in the C-LPS group compared to those in the C-CON, N-CON, and N-LPS groups. The ALP values in the C-LPS group were higher than those in the C-CON group (*P* < 0.05), but there was no significant difference between the C-LPS, N-CON, and N-LPS groups.

**Table 3:** Effects of maternal nucleotide supplementation on the serum parameters in lipopolysaccharide-challenged newborn piglets^1^.

**Items^2^**	**Group** ^**3**^				***P*** **-values** ^**4**^		
	**C-CON**	**C-LPS**	**N-CON**	**N-LPS**	**Diet**	**LPS**	**D × L**
hs-CRP, ug/mL	28.79 ± 0.71^b^	34.15 ± 0.88^a^	27.96 ± 0.63^b^	29.67 ± 0.64^b^	0.001	<0.001	0.020
C3, μg/mL	276.39 ± 3.14	223.94 ± 3.02	302.99 ± 8.66	234.66 ± 5.25	0.003	<0.001	0.165
C4, μg/mL	31.95 ± 0.90	32.39 ± 0.89	34.31 ± 0.82	33.81 ± 0.80	0.038	0.971	0.589
AST, U/L	9.32 ± 0.91	10.29 ± 0.81	9.80 ± 0.49	9.54 ± 1.04	0.874	0.673	0.473
ALT, U/L	5.80 ± 1.47	10.30 ± 1.00	5.80 ± 1.27	7.49 ± 1.53	0.306	0.031	0.304
ALP, U/L	85.14 ± 1.78^b^	94.02 ± 2.49^a^	90.90 ± 2.22^ab^	87.43 ± 2.34^ab^	0.855	0.239	0.012

^1^Data were expressed as means with their standard errors, n = 6.

^2^hs-CRP, hypersensitive C-reactive protein; C3, complement C3; C4, complement C4; AST, aspartate aminotransferase; ALT, alanine aminotransferase; ALP, alkaline phosphatases.

^3^C-CON, piglets were from the sows fed with basal diets and were challenged by sterile saline; C-LPS, piglets were from the sows fed with basal diets and were challenged by LPS; N-CON, piglets were from the sows fed with basal diets containing nucleotides and were challenged by sterile saline; N-LPS, piglets were from the sows fed with basal diets containing nucleotides and were challenged by LPS.

^4^Diet (D), the effect of maternal diet; LPS (L), the effect of LPS challenge; D × L, the interaction between maternal diet and LPS challenge.

a,b Different superscript letters of the mean in the same row represent a significant difference (*P* < 0.05).

### Serum cytokine concentrations analysis

Piglets challenged with LPS had higher serum IL-1β, IL-6, TNF-α, and COX-2 levels than those injected with sterile saline (*P* < 0.05), as shown in Table 4. The maternal NT diet reduced serum IL-1β and COX-2 levels in newborn piglets (*P* < 0.05) and tended to decrease the serum IL-6 concentrations (*P* = 0.098). Notably, the maternal NT diet interacted with the LPS challenge for IL-10 content (*P* < 0.05). The N-CON group had higher serum IL-10 levels (*P* < 0.05) than the C-CON, C-LPS, and N-LPS groups.

**Table 4:** Effects of maternal nucleotide supplementation on the serum cytokine concentrations in lipopolysaccharide-challenged newborn piglets^1^.

**Items^2^**	**Group** ^**3**^				***P*** **-values** ^**4**^		
	**C-CON**	**C-LPS**	**N-CON**	**N-LPS**	**Diet**	**LPS**	**D × L**
IL-1β, pg/mL	475.22 ± 9.79	530.88 ± 14.01	421.52 ± 6.40	494.00 ± 11.22	<0.001	<0.001	0.442
IL-10, pg/mL	547.30 ± 11.60^b^	514.74 ± 9.87^c^	622.30 ± 8.40^a^	538.16 ± 9.10^bc^	<0.001	<0.001	0.016
IL-6, ng/mL	3.00 ± 0.07	3.35 ± 0.13	2.96 ± 0.08	3.05 ± 0.10	0.098	0.036	0.209
TNF-α, ng/mL	786.20 ± 18.95	827.20 ± 11.71	770.92 ± 19.78	804.97 ± 18.24	0.296	0.044	0.844
COX-2, ng/mL	51.89 ± 0.82	61.15 ± 0.94	48.77 ± 1.12	56.55 ± 0.82	0.001	<0.001	0.438

^1^Data were expressed as means with their standard errors, n = 6.

^2^IL-1β, interleukin 1 β; IL-10, interleukin 10; IL-6, interleukin 6; TNF-α, tumor necrosis factor α; COX-2, cyclooxygenase 2.

^3^C-CON, piglets were from the sows fed with basal diets and were challenged by sterile saline; C-LPS, piglets were from the sows fed with basal diets and were challenged by LPS; N-CON, piglets were from the sows fed with basal diets containing nucleotides and were challenged by sterile saline; N-LPS, piglets were from the sows fed with basal diets containing nucleotides and were challenged by LPS.

^4^Diet (D), the effect of maternal diet; LPS (L), the effect of LPS challenge; D × L, the interaction between maternal diet and LPS challenge.

a,b,c Different superscript letters of the mean in the same row represent a significant difference (*P* < 0.05).

### Intestinal morphology and globet cell density

**Figure 1:**
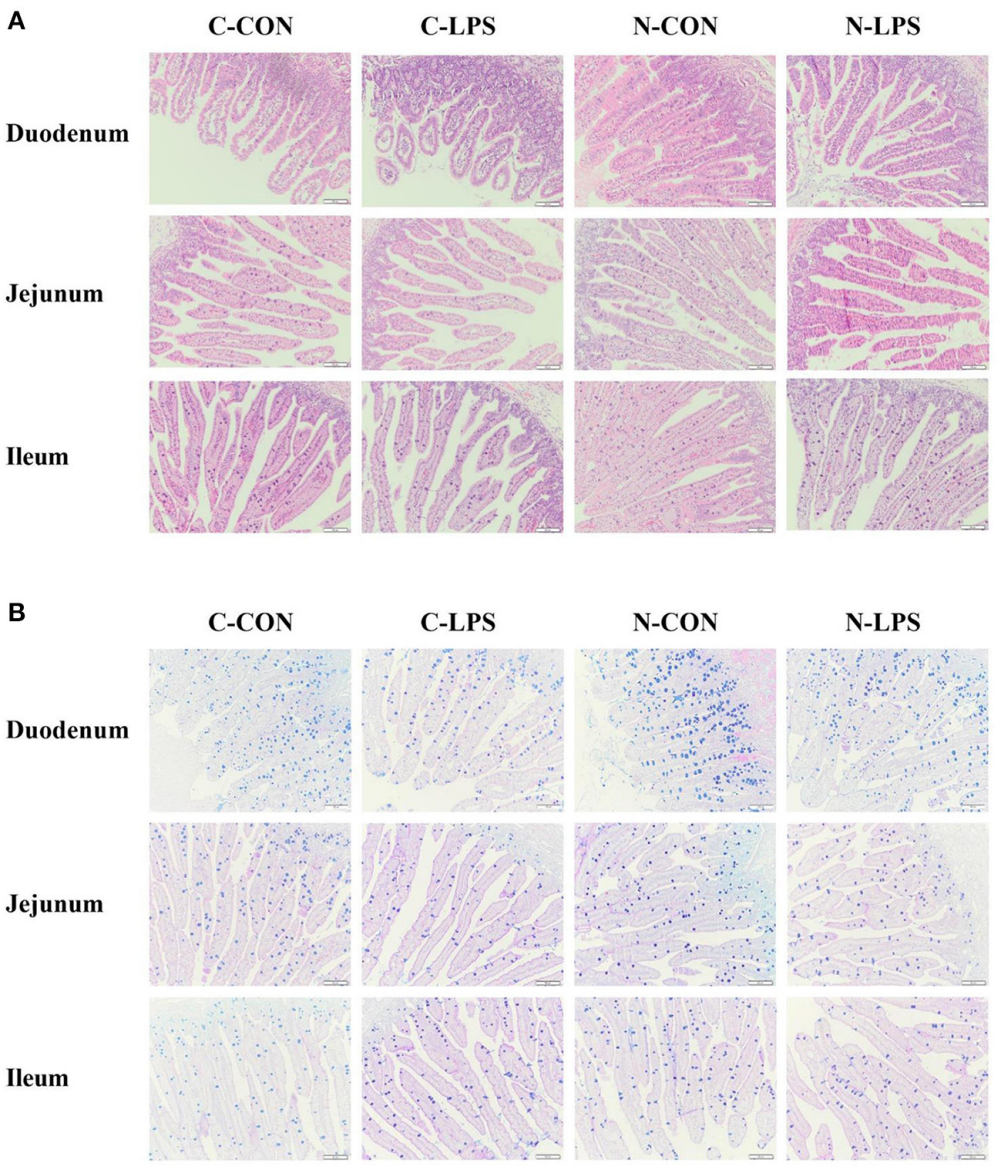
Effects of maternal nucleotide supplementation on the intestinal morphology and globet cells density in lipopolysaccharide-challenged newborn piglets. **(A)** Hematoxylin and eosin (H&E) staining on the small intestine. **(B)** Alcian blue and periodic acid-schiff (AB-PAS) staining on the small intestine. Scale bar represents 100 μm. C-CON, piglets were from the sows fed with basal diets and were challenged by sterile saline; C-LPS, piglets were from the sows fed with basal diets and were challenged by LPS; N-CON, piglets were from the sows fed with basal diets containing nucleotides and were challenged by sterile saline; N-LPS, piglets were from the sows fed with basal diets containing nucleotides and were challenged by LPS.

To evaluate the intestinal morphology and globet cell density, VH, CD, VCR, and the density of GCs in the small intestine (Figure 1) were measured. The results in Table 5 showed that maternal NT supplementation increased the VH and VCR (*P* < 0.05) in the duodenum, increased the CD in the jejunum (*P* < 0.05), and tended to decrease the CD in the duodenum (*P* = 0.070). Compared to piglets injected with sterile saline, piglets exposed to LPS treatment had lower VH and VCR in the duodenum (*P* < 0.05) and higher CD in the duodenum, as well as lower CD jejunum (*P* < 0.05). An interaction was observed between the maternal diet and LPS challenge on the VH in the jejunum and ileum. In the jejunum, the VH in the C-LPS group was lower than that in the other three groups (*P* < 0.05). In the ileum, the N-CON and N-LPS groups had higher VH than the C-CON and C-LPS groups (*P* < 0.05), but there was no significant difference between the N-CON and N-LPS groups.

**Table 5:** Effects of maternal nucleotide supplementation on the intestinal morphology and globet cells density in lipopolysaccharide-challenged newborn piglets^1^.

**Items^2^**	**Group** ^**3**^				***P*** **-values** ^**4**^		
	**C-CON**	**C-LPS**	**N-CON**	**N-LPS**	**Diet**	**LPS**	**D × L**
**Duodenum**							
VH (μm)	418.32 ± 10.23	398.35 ± 8.56	466.01 ± 7.51	431.94 ± 3.05	0.001	0.009	0.393
CD (μm)	148.87 ± 9.70	176.79 ± 4.05	144.80 ± 5.83	154.92 ± 3.10	0.070	0.016	0.189
VCR	2.95 ± 0.22	2.32 ± 0.10	3.37 ± 0.22	2.87 ± 0.11	0.024	0.012	0.712
GCs (n/100 μm VH)	3.49 ± 0.09	3.02 ± 0.17	3.90 ± 0.13	3.46 ± 0.17	0.018	0.013	0.905
**Jejunum**							
VH (μm)	701.38 ± 7.00^c^	654.72 ± 5.31^d^	748.19 ± 5.10^a^	727.76 ± 2.67^b^	<0.001	<0.001	0.037
CD (μm)	111.62 ± 0.44	101.02 ± 3.68	114.60 ± 2.59	110.04 ± 2.04	0.042	0.016	0.258
VCR	6.49 ± 0.12	6.59 ± 0.30	6.71 ± 0.12	6.83 ± 0.07	0.220	0.551	0.953
GCs (n/100 μm VH)	2.26 ± 0.14	1.75 ± 0.05	2.37 ± 0.06	2.11 ± 0.22	0.125	0.021	0.387
**Ileum**							
VH (μm)	685.93 ± 3.14^b^	676.63 ± 3.91^b^	704.22 ± 3.28^a^	713.30 ± 1.01^a^	<0.001	0.972	0.017
CD (μm)	85.63 ± 3.32	90.13 ± 1.85	87.56 ± 5.88	94.11 ± 5.70	0.531	0.256	0.825
VCR	8.25 ± 0.33	7.75 ± 0.05	8.57 ± 0.56	7.82 ± 0.42	0.627	0.143	0.754
GCs (n/100 μm VH)	1.60 ± 0.05	1.62 ± 0.07	2.53 ± 0.23	2.06 ± 0.12	0.001	0.136	0.109

^1^Data were expressed as means with their standard errors, n = 6.

^2^VH, villus height; CD, crypt depth; VCR, villus height to crypt depth ratio; GCs, the number of globet cells per 100 μm villus height.

^3^C-CON, piglets were from the sows fed with basal diets and were challenged by sterile saline; C-LPS, piglets were from the sows fed with basal diets and were challenged by LPS; N-CON, piglets were from the sows fed with basal diets containing nucleotides and were challenged by sterile saline; N-LPS, piglets were from the sows fed with basal diets containing nucleotides and were challenged by LPS.

^4^Diet (D), the effect of maternal diet; LPS (L), the effect of LPS challenge; D × L, the interaction between maternal diet and LPS challenge.

a−d Different superscript letters of the mean in the same row represent a significant difference (*P* < 0.05).

Compared to the piglets treated with sterile saline, the density of GCs in the duodenum and jejunum of the pigs challenged with LPS decreased significantly (*P* < 0.05). The maternal NT diet increased the density of piglets' GCs in the duodenum and ileum (*P* < 0.05), but no effect of interaction between maternal diet and LPS challenge was observed on the density of GCs.

### Jejunal and ileal cytokine levels

**Figure 2:**
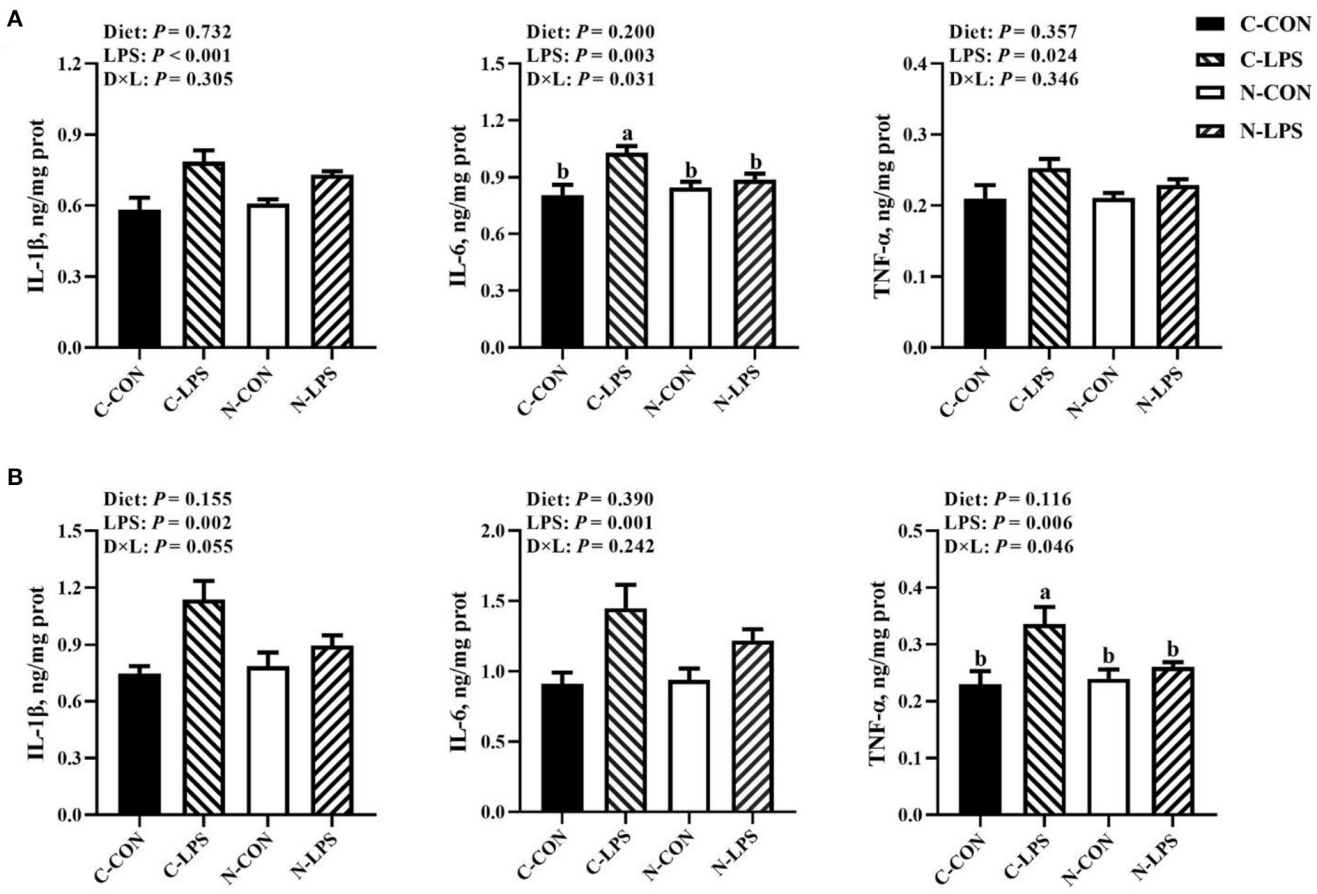
Effects of maternal nucleotide supplementation on cytokine levels in jejunum and ileum in lipopolysaccharide-challenged newborn piglets. **(A)** Cytokine levels (IL-1β, IL-6, TNF-α) in jejunum. **(B)** Cytokine levels (IL-1β, IL-6, TNF-α) in ileum. Results were expressed as means with their standard errors, *n* = 6; ^a,b^Means of the same parameter with different superscripts are significantly different (*P* < 0.05). IL-1β, interleukin-1β; IL-6, interleukin-6; TNF-α, tumor necrosis factor-α. C-CON, piglets were from the sows fed with basal diets and were challenged by sterile saline; C-LPS, piglets were from the sows fed with basal diets and were challenged by LPS; N-CON, piglets were from the sows fed with basal diets containing nucleotides and were challenged by sterile saline; N-LPS, piglets were from the sows fed with basal diets containing nucleotides and were challenged by LPS.

As shown in Figure 2, the concentrations of IL-1β, IL-6, and TNF-α in the jejunum and ileum were increased (*P* < 0.05) when newborn piglets were stimulated by LPS. An interaction was observed between the maternal diet and LPS challenge on jejunal IL-6 contents (*P* < 0.05) and ileal TNF-α contents (*P* < 0.05). Compared to the C-LPS group, the concentrations of IL-6 in the jejunum were lower in the C-CON, N-CON, and N-LPS groups (*P* < 0.05). The ileal TNF-α levels in the C-LPS group were higher than those in the other three groups (*P* < 0.05).

### Intestinal inflammation-related gene expression

**Figure 3:**
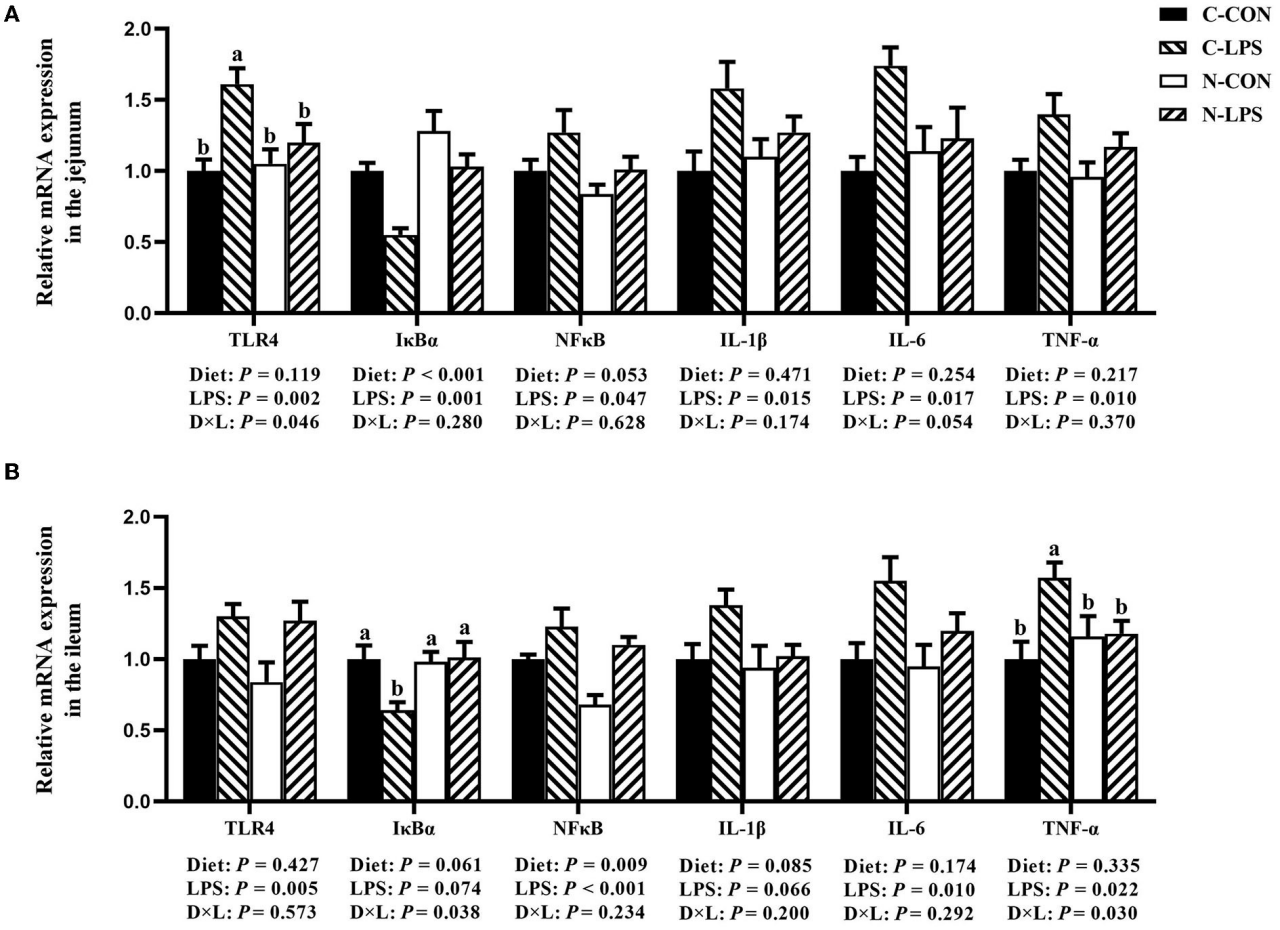
Effects of maternal nucleotide supplementation on inflammation-related gene expression in jejunum and ileum in lipopolysaccharide-challenged newborn piglets. **(A)** Relative mRNA expression in jejunum. **(B)** Relative mRNA expression in ileum. Results were expressed as means with their standard errors, *n* = 6; ^a,b^Means of the same parameter with different superscripts are significantly different (*P* < 0.05). TLR4, toll-like receptor 4; NFκB, nuclear factor kappa B; IκBα, nuclear factor kappa B inhibitor alpha; IL-1β, interleukin-1β; IL-6, interleukin-6; TNF-α, tumor necrosis factor-α. C-CON, piglets were from the sows fed with basal diets and were challenged by sterile saline; C-LPS, piglets were from the sows fed with basal diets and were challenged by LPS; N-CON, piglets were from the sows fed with basal diets containing nucleotides and were challenged by sterile saline; N-LPS, piglets were from the sows fed with basal diets containing nucleotides and were challenged by LPS.

According to the results shown in Figure 3, the maternal NT supplementation increased the mRNA expression of IκBα in the jejunum (*P* < 0.05) and decreased the ileal NFκB expression (*P* < 0.05). In addition, the mRNA expression levels of the jejunal NFκB (*P* = 0.053) and ileal IL-1β (*P* = 0.085) had decreasing tendencies in maternal NT supplementation groups. The jejunal and ileal mRNA expression levels measured in this experiment increased in newborn piglets after receiving LPS treatment (*P* < 0.05), except for IκBα and ileal IL-1β. Furthermore, there was an interaction observed between the maternal diet and LPS challenge for the jejunal TLR4 mRNA expression and the ileal IκBα and TNF-α mRNA expressions (*P* < 0.05). Compared to the C-LPS group, the mRNA expression of TLR4 in the jejunum was lower in the C-CON, N-CON, and N-LPS groups (*P* < 0.05). The ileum TNF-α expression level was higher (*P* < 0.05) in the C-LPS group than in other groups. But the IκBα expression level in the C-LPS group was lower (*P* < 0.05) than in other three groups.

### Intestinal inflammation-related protein expression

**Figure 4:**
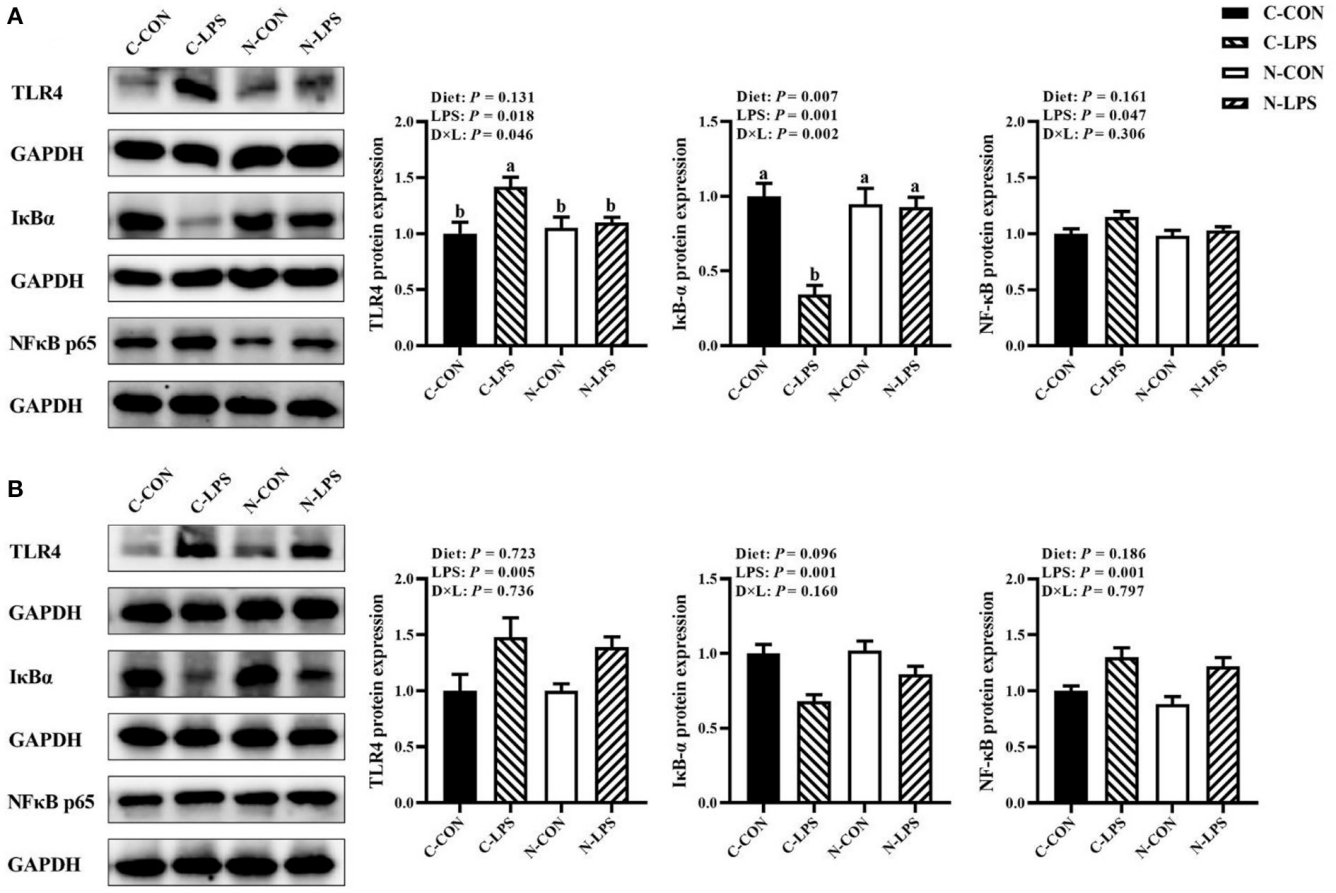
Effects of maternal nucleotide supplementation on inflammation-related protein expression in jejunum and ileum in lipopolysaccharide-challenged newborn piglets. **(A)** Inflammation-related protein expression in jejunum. **(B)** Inflammation-related protein expression in ileum. Results were expressed as means with their standard errors, *n* = 6; ^a,b^Means of the same parameter with different superscripts are significantly different (*P* < 0.05). TLR4, toll-like receptor 4; NFκB p65, nuclear factor kappa B p65 subunit; IκBα, nuclear factor kappa B inhibitor alpha; GAPDH, glyceraldehyde-3-phosphate dehydrogenase. C-CON, piglets were from the sows fed with basal diets and were challenged by sterile saline; C-LPS, piglets were from the sows fed with basal diets and were challenged by LPS; N-CON, piglets were from the sows fed with basal diets containing nucleotides and were challenged by sterile saline; N-LPS, piglets were from the sows fed with basal diets containing nucleotides and were challenged by LPS.

As shown in Figure 4, when newborn piglets were challenged with LPS, the protein expressions of TLR4 and NFκB p65 in the jejunum and ileum were increased (*P* < 0.05), and the maternal NT diet tended to increase the expression level of IκBα protein in the ileum (*P* = 0.096). An interaction was observed between the maternal diet and LPS challenge on TLR4 and IκBα protein expressions in the jejunum (*P* < 0.05). The jejunal protein expression of TLR4 was higher, and the IκBα protein expression was lower in the C-LPS group than those in the other three groups (*P* < 0.05).

## Discussion

During gestation, sows receive nutrients from their daily diets and provide the fetal piglets with nutrients that are necessary for development. However, newborn piglets have immature intestinal morphology and immune function, and the environmental stressors may affect physiological functions of piglets. NTs, as conditionally essential nutrients, have been used in sows' diets and had demonstrated its ability to modulate sow milk composition and improve weaning piglet numbers (9, 10). These results suggested that feeding exogenous NTs to sows may have some beneficial effects. Therefore, in the current study, we investigated the effects of maternal NT supplementation on the intestinal morphology and immune function of newborn piglets, as well as whether maternal NT supplementation could enhance the resistance of newborn piglets to LPS.

ALT, AST, and ALP are mainly present in the liver, and these enzymes are released into the blood when liver cells are damaged. Therefore, their changes in serum always serve as classic indicators of liver injury (21). In addition, CRP is also expressed and secreted by the liver. As an acute-phase protein, the serum levels of CRP can increase rapidly in response to tissue injury or infection (22). In this study, LPS injection induced elevated serum ALT and ALP concentrations in newborn piglets; the results agreed with many previous studies (23–25). The serum hs-CRP levels in the N-CON and N-LPS groups were lower than the C-LPS group. Exogenous NTs can modulate hepatocyte growth and regeneration (26) and improve liver function to alleviate liver injury induced by D-galactosamine in rats (27). Some studies have demonstrated the potential anti-bacterial and anti-infective effects of NTs (12, 28). However, there are few reports on the effects of exogenous NTs on the liver health in piglets. Combined with the results of this experiment, we speculated that feeding exogenous NTs to sows may promote the liver development in piglets, and increase the resistance to infection induced by LPS. Additionally, LPS can activate monocytes and macrophages to produce different cytokines to induce an inflammatory response. Based on their role in inflammation, some cytokines are known as pro-inflammatory cytokines because they are involved in promoting inflammation (TNF-α, IL-1β, IL-6, etc), and other cytokines that suppress inflammation are known as anti-inflammatory cytokines (IL-10, IL-4, etc) (29). COX-2, induced by pro-inflammatory cytokines, is an inducible pro-inflammatory enzyme that participates in the inflammatory response (30). The stress response caused by LPS has been reported to result in increased levels of pro-inflammatory cytokines in piglets (31, 32). We found out that the levels of IL-1β, IL-6, TNF-α, and COX-2 in the serum of piglets stimulated by LPS were significantly increased, while maternal NT supplementation decreased the IL-1β and COX-2 levels in the serum of piglets. Simultaneously, serum IL-10 was significantly improved by maternal NT supplementation. Some pro-inflammatory cytokines maintained at appropriate levels are beneficial for resisting infection; however, overstimulation of the immune system have deleterious effects (33). The published article mentioned the immune regulation function of NTs which could increase the concentration of IL-10 and lower the IL-1β in piglets' serum under stress conditions such as weaning (34). In this study, the increase in serum IL-10 levels induced by maternal NT supplementation demonstrates the anti-inflammatory properties of NTs and may influence the serum pro-inflammatory cytokine levels in newborn pigs.

The small intestine is the primary organ responsible for absorption and transportation, and a well-developed intestinal structure is the foundation for nutrient utilization and healthy growth in piglets. VH, CD, and VCR are always used to evaluate the digestive and absorption functions of the small intestine (35). In the current trial, the maternal NT diet in late gestation significantly increased the VH in the small intestine of newborn piglets, which was in agreement with the previous research (10). In the small intestine, the villi, the essential structure for absorbing nutrients and fluids, are composed of intestinal epithelial cells. Intestinal epithelial cells have a rapid renewal rate but are always poor in synthesizing NTs by endogenous pathways (3, 36). The final weeks before parturition are critical for the intestinal development and the maturation of the intestinal digestive function (37). During this stage, fetal pigs mainly obtain the nutrients needed for development from sows through the placenta, which includes a variety of nutrient transporters, some of which can transport NTs (38, 39). Hence, we hypothesized that sows fed a basic diet containing NTs can provide NTs to piglets through the placenta and promote intestinal development in fetal pigs. In addition, LPS is present in the cell wall of Gram-negative bacteria, and could result in morphological changes in intestine, such as submucosal edema, epithelial vacuolation, and necrosis (40, 41). Previous studies have also reported that LPS causes a decrease in the height of the intestinal villi (35, 42); however, our study showed that the VH of the jejunum and ileum in the N-LPS group was higher than the C-LPS group. This difference might have resulted from the superior developed intestinal structure caused by maternal NT supplementation. Furthermore, the surface of the intestinal epithelium in contact with the external environment is protected by mucins. These components can prevent the attachment of microbes to the intestinal epithelium, and are mainly synthesized and secreted by goblet cells (43). However, previous studies have demonstrated that LPS challenge could impaire goblet cell proliferation and affect the density of goblet cells in intestine (43, 44). In this experiment, we have found that LPS reduced the density of intestinal goblet cells, but maternal NT supplementation significantly increased the density of goblet cells in the duodenum and ileum of piglets. The increase may be related to the proliferation of goblet cells in fetal pigs promoted by maternal NT supplementation. Nevertheless, the exact mechanism of regulation of the intestinal goblet cells density in newborn piglets by maternal NT supplementation or LPS treatment requires further investigation.

Newborn piglets are severely immunodeficient at birth and require passive immunity from ingested breast milk. And this immaturity of the immune function in newborn piglets does not improve effectively until the piglets produce their own immunoglobulin at 3–4 weeks old (45). In the circumstances, the innate immune system that the piglets possess from birth builds up the defenses to protect piglets from microorganisms and antigens. As a part of innate immunity, the complement system participates in immune defense, and the increased secretion of C3 and C4 generally indicates that the immune response is strengthened and disease resistance is enhanced (46). In the present trial, maternal NT supplementation significantly increased the serum C3 and C4 concentrations, suggesting the effect of maternal NT supplementation on improving the innate immunity of newborn pigs. The intestine plays an important role in digesting and absorbing nutrients and compensating the body's immune system. The innate immune response in the piglets' intestine can assist in recognizing antigens by identifying the highly conserved structures present in large groups of microorganisms instead of recognizing each (47). These conserved structures of microorganisms are known as pathogen-associated molecular patterns, and the receptors used to recognize these structures in the innate immune system are called pattern-recognition receptors (PRRs) (48). TLR4, a receptor studied extensively in the toll family, belongs to the PRRs family and is known as an LPS receptor. Upon LPS recognition, TLR4 is activated and initiates its downstream signaling pathways. The NFκB/IκBα pathway is a major signaling pathway activated by TLR4 combined with LPS. Here, we further investigated the regulatory effects of maternal NT supplementation on the expression of mRNA and proteins involved in the TLR4/IκBα/NFκB pathway in LPS-challenged newborn piglets. The results showed that LPS challenge increased the mRNA expression of TLR4 and NFκB and decreased IκBα mRNA expression in the jejunum and ileum of piglets, the expression of the corresponding proteins also showed the same trend. These results are in line with those of previous studies (49, 50). Under normal conditions, NFκB forms a complex with IκBα and remains inactive in the cytoplasm. After stimulation with LPS, IκBα is phosphorylated and degraded, and activated NFκB is translocated to the nucleus and binds to DNA (51). Notably, maternal NT supplementation suppressed the increase in TLR4 mRNA and protein expression and maintained the IκBα mRNA and protein at normal expression level in the jejunum of piglets treated by LPS; at the same time, the addition of NTs to the sow diets had a facilitative effect on the expression of IκBα protein in ileum. Additionally, the concentrations of IL-1β, TNF-α, and IL-6 in the jejunum and ileum were increased after newborn pigs were stimulated by LPS, but maternal NT supplementation inhibited the increase in IL-6 content in the jejunum and the TNF-α content in the ileum. From a molecular point of view, the mRNA expression of IL-1β, TNF-α, and IL-6 was significantly increased after the newborn pigs were treated with LPS, but the maternal NT supplementation could inhibit the increase of IL-1β and TNF-α. A previous study reported that LPS treatment resulted in an increase of cytokines (TNF-α, IL-1β, and IL-6) levels and heightened the phosphorylation of NFκB in mice (52). However, in the present study, the LPS-induced negative changes in key genes and proteins of the TLR4/IκBα/NFκB signaling pathway and genes related to inflammatory factors regulated by this pathway were improved by maternal NT supplementation. Taken together, our findings suggested that maternal NT supplementation alleviates LPS-induced intestinal inflammation in piglets, which may be related to the regulation of the TLR4/IκBα/NFκB pathway.

## Conclusion

Adding exogenous NTs to the basal diet of sows during late gestation can promote intestinal development in newborn piglets, enhance the resistance of piglets to LPS-induced stress responses, and alleviate intestinal damage induced by LPS injection. The results also suggest that maternal NT addition could enhance the innate immune function of newborn piglets and alleviate the LPS-induced intestinal inflammatory response by regulating the TLR4/IκBα/NFκB signaling pathway.

## Data availability statement

The raw data supporting the conclusions of this article will be made available by the authors, without undue reservation.

## Ethics statement

The animal study was reviewed and approved by the Nanjing Agricultural University Institutional Animal Care and Use Committee.

## Author contributions

QL, KB, IH, and TW conceived and designed the experiments. QL, IH, and KB prepared material, collected data, and revised the manuscript. QL analyzed the data and prepared and drafted the manuscript. All authors have read and agreed to the published version of the manuscript.

## Funding

This study was financially supported by the National Natural Science Foundation of China (Grant No. 32172775).

## Conflict of interest

Author IH was employed by Anyou Biotechnology Group Co., Ltd. The remaining authors declare that the research was conducted in the absence of any commercial or financial relationships that could be construed as a potential conflict of interest.

## Publisher's note

All claims expressed in this article are solely those of the authors and do not necessarily represent those of their affiliated organizations, or those of the publisher, the editors and the reviewers. Any product that may be evaluated in this article, or claim that may be made by its manufacturer, is not guaranteed or endorsed by the publisher.

## References

[1] Hess JR Greenberg NA. The role of nucleotides in the immune and gastrointestinal systems. Nutr Clin Prac. (2012) 27:281–94. 10.1177/0884533611434933 22392907

[2] Buren C Rudolph F. Dietary nucleotides: a conditional requirement. Nutrition. (1997) 13:470–2. 10.1016/S0899-9007(97)00103-2 9225342

[3] Uauy R Quan R Gil A. Role of nucleotides in intestinal development and repair: implications for infant nutrition. J Nutr. (1994) 124:1436S–41S. 10.1093/jn/124.suppl_8.1436S 8064399

[4] Carver JD Walker WA. The role of nucleotides in human nutrition. J Nutr Biochem. (1995) 6:58–72. 10.1016/0955-2863(94)00019-I

[5] Mateo CD. Aspects of Nucleotide Nutrition in Pigs. Brookings, South Dakota USA: South Dakota State University (2005).

[6] Yang J Upadhaya SD Kim IH. Effects of nucleotide supplementation to corn–soybean meal-based diet on growth performance, fecal microflora, and blood profiles of sows and performance of suckling piglets. Can J Anim Sci. (2019) 99:754–63. 10.1139/cjas-2018-0222

[7] Yang J Kim IH. Effects of nucleotide supplementation on growth performance, nutrient digestibility, and immune blood profiles related to foot-and-mouth disease in vaccinated growing pigs. Can J Anim Sci. (2019) 99:326–31. 10.1139/cjas-2018-0087

[8] Martinez-Puig D Manzanilla EG Morales J Borda E Pérez JF Piñeiro C. Dietary nucleotide supplementation reduces occurrence of diarrhoea in early weaned pigs. Livest Sci. (2007) 108:276–9. 10.1016/j.livsci.2007.01.099

[9] Tan C Ji Y Zhao X Xin Z Li J Huang S. Effects of dietary supplementation of nucleotides from late gestation to lactation on the performance and oxidative stress status of sows and their offspring. Anim Nutr. (2021) 7:111–8. 10.1016/j.aninu.2020.10.004 33997338 PMC8110849

[10] Gao L Xie C Liang X Li Z Li B Wu X. Yeast-based nucleotide supplementation in mother sows modifies the intestinal barrier function and immune response of neonatal pigs. Anim Nutr. (2021) 7:84–93. 10.1016/j.aninu.2020.06.009 33997335 PMC8110885

[11] Andersen IL Tajet GM Haukvik IA Kongsrud S Bøe KE. Relationship between postnatal piglet mortality, environmental factors and management around farrowing in herds with loose-housed, lactating sows. Acta Agric Scand Sect A. (2007) 57:38–45. 10.1080/09064700601159626

[12] Valini GA Duarte MS Calderano AA Teixeira LM Rodrigues GA Fernandes KM. Dietary nucleotide supplementation as an alternative to in-feed antibiotics in weaned piglets. Animal. (2021) 15:100021. 10.1016/j.animal.2020.100021 33573936

[13] Bueno J Torres M Almendros A Carmona R Nunez MC Rios A. Effect of dietary nucleotides on small intestinal repair after diarrhoea. Histological and ultrastructural changes. Gut. (1994) 35:926–33. 10.1136/gut.35.7.926 8063220 PMC1374839

[14] Tran AX Whitfield C. Lipopolysaccharides (endotoxins). In:Schaechter M, editor. Encyclopedia of Microbiology. 3rd edition. Oxford: Academic Press. (2009). p. 513–28. 10.1016/B978-012373944-5.00196-6

[15] Han H Zhang J Chen Y Shen M Yan E Wei C. Dietary taurine supplementation attenuates lipopolysaccharide-induced inflammatory responses and oxidative stress of broiler chickens at an early age. J Anim Sci. (2020) 98:skaa311. 10.1093/jas/skaa311 32954422 PMC7584273

[16] National Research Council. Nutrient Requirements of Swine. 11th rev ed. Washington, DC: Nat Acad Press (2012).

[17] Geng S Cheng S Li Y Wen Z Ma X Jiang X. Fecal microbiota transplantation reduces susceptibility to epithelial injury and modulates tryptophan metabolism of microbial community in a piglet model. J Crohns Colitis. (2018) 12:1359–74. 10.1093/ecco-jcc/jjy103 30010734

[18] Yun Y Ji S Yu G Jia P Niu Y Zhang H. Effects of Bacillus subtilis on jejunal integrity, redox status, and microbial composition of intrauterine growth restriction suckling piglets. J Anim Sci. (2021) 99:skab255. 10.1093/jas/skab255 34473279 PMC8493892

[19] Li Y Zhang H Su W Ying Z Chen Y Zhang L. Effects of dietary bacillus amyloliquefaciens supplementation on growth performance, intestinal morphology, inflammatory response, and microbiota of intra-uterine growth retarded weanling piglets. J Anim Sci Biotechnol. (2018) 9:22. 10.1186/s40104-018-0236-2 29564121 PMC5848560

[20] Schmittgen TD Livak KJ. Analyzing real-time PCR data by the comparative CT method. Nat Protoc. (2008) 3:1101–8. 10.1038/nprot.2008.73 18546601

[21] Senior JR. Alanine aminotransferase: a clinical and regulatory tool for detecting liver injury–past, present, and future. Clin Pharmacol Ther. (2012) 92:332–9. 10.1038/clpt.2012.108 22871997

[22] Yao Z Zhang Y Wu H. Regulation of C-reactive protein conformation in inflammation. Inflamm Res. (2019) 68:815–23. 10.1007/s00011-019-01269-1 31312858

[23] Zhang Y Deng ZX He ML Pastor JJ Tedo G Liu JX. Olive oil cake extract stabilizes the physiological condition of lipopolysaccharide-challenged piglets by reducing oxidative stress and inflammatory responses and modulating the ileal microbiome. Food Funct. (2021) 12:10171–83. 10.1039/D0FO03012K 34529747

[24] Wang K Zhang H Han Q Lan J Chen G Cao G. Effects of astragalus and ginseng polysaccharides on growth performance, immune function and intestinal barrier in weaned piglets challenged with lipopolysaccharide. J Anim Physiol Anim Nutr. (2020) 104:1096–105. 10.1111/jpn.13244 31724241

[25] Duan Y Song B Zheng C Zhong Y Guo Q Zheng J. Dietary beta-hydroxy beta-methyl butyrate supplementation alleviates liver injury in lipopolysaccharide-challenged piglets. Oxid Med Cell Longev. (2021) 2021:5546843. 10.1155/2021/5546843 33868570 PMC8035022

[26] Novak DA Carver JD Barness LA. Dietary nucleotides affect hepatic growth and composition in the weanling mouse. J Parenter Enteral Nutr. (1994) 18:62–6. 10.1177/014860719401800162 8164306

[27] Ogoshi S Iwasa M Kitagawa S Ohmori Y Mizobuchi S Iwasa Y. Effects of total parenteral nutrition with nucleoside and nucleotide mixture on D-galactosamine-induced liver injury in rats. J Parenter Enteral Nutr. (1988) 12:53–7. 10.1177/014860718801200153 3125356

[28] Sauer N Bauer E Vahjen W Zentek J Mosenthin R. Nucleotides modify growth of selected intestinal bacteria in vitro. Livest Sci. (2010) 133:161–3. 10.1016/j.livsci.2010.06.053

[29] Rossol M Heine H Meusch U Quandt D Klein C Sweet MJ. LPS-induced cytokine production in human monocytes and macrophages. Crit Rev Immunol. (2011) 31:379–446. 10.1615/CritRevImmunol.v31.i5.20 22142165

[30] Prabhakaran J Molotkov A Mintz A Mann JJ. Progress in pet imaging of neuroinflammation targeting cox-2 enzyme. Molecules. (2021) 26:3208. 10.3390/molecules26113208 34071951 PMC8198977

[31] Rossol M Heine H Meusch U Quandt D Klein C Sweet MJ. The regulation effects of danofloxacin on pig immune stress induced by LPS. Res Vet Sci. (2017) 110:65–71. 10.1016/j.rvsc.2016.11.005 28159239

[32] Xu B Yan Y Yin B Zhang L Qin W Niu Y. Dietary glycyl-glutamine supplementation ameliorates intestinal integrity, inflammatory response, and oxidative status in association with the gut microbiota in LPS-challenged piglets. Food Funct. (2021) 12:3539–51. 10.1039/D0FO03080E 33900316

[33] Li H Zhao P Lei Y Li T Kim I. Response to an Escherichia coli K88 oral challenge and productivity of weanling pigs receiving a dietary nucleotides supplement. J Anim Sci Biotechnol. (2015) 6:49. 10.1186/s40104-015-0049-5 26635958 PMC4668697

[34] Superchi P Saleri R Borghetti P De Angelis E Ferrari L Cavalli V. Effects of dietary nucleotide supplementation on growth performance and hormonal and immune responses of piglets. Animal. (2011) 6:902–8. 10.1017/S1751731111002473 22558960

[35] Liu Y Huang J Hou Y Zhu H Zhao S Ding B. Dietary arginine supplementation alleviates intestinal mucosal disruption induced by *Escherichia coli* lipopolysaccharide in weaned pigs. Br J Nutr. (2008) 100:552–60. 10.1017/S0007114508911612 18275628

[36] Savaiano DA Clifford AJ. Adenine, the precursor of nucleic acids in intestinal cells unable to synthesize purines *de novo*. J Nutr. (1981) 111:1816–22. 10.1093/jn/111.10.1816 7288504

[37] Buccigrossi V Giannattasio A Armellino C Vecchio AL Caiazzo MA Guarino A. The functional effects of nutrients on enterocyte proliferation and intestinal ion transport in early infancy. Early Hum Dev. (2010) 86(1 Supplement):55–7. 10.1016/j.earlhumdev.2010.01.008 20153590

[38] Nogues P Dos Santos E Couturier-Tarrade A Berveiller P Arnould L Lamy E. Maternal obesity influences placental nutrient transport, inflammatory status, and morphology in human term placenta. J Clin Endocrinol Metab. (2020) 106:1880–96. 10.1210/clinem/dgaa660 32936881

[39] Young JD. The SLC28 (CNT) and SLC29 (ENT) nucleoside transporter families: a 30-year collaborative odyssey. Biochem Soc Trans. (2016) 44:869–76. 10.1042/BST20160038 27284054

[40] Yi D Hou Y Wang L Zhao D Ding B Wu T. Gene expression profiles in the intestine of lipopolysaccharide-challenged piglets. FBL. (2016) 21:487–501. 10.2741/4404 26709789

[41] Williams JM Duckworth CA Watson AJM Frey MR Miguel JC Burkitt MD. A mouse model of pathological small intestinal epithelial cell apoptosis and shedding induced by systemic administration of lipopolysaccharide. Dis Models Mech. (2013) 6:1388–99. 10.1242/dmm.013284 24046352 PMC3820262

[42] Xiao X Cheng Y Fu J Lu Z Wang F Jin M. Gut immunity and microbiota dysbiosis are associated with altered bile acid metabolism in lps-challenged piglets. Oxid Med Cell Longev. (2021) 2021:6634821. 10.1155/2021/6634821 33833852 PMC8018853

[43] Birchenough GM Johansson ME Gustafsson JK Bergström JH Hansson G. New developments in goblet cell mucus secretion and function. Mucosal Immunol. (2015) 8:712–9. 10.1038/mi.2015.32 25872481 PMC4631840

[44] Li C Ma D Zhou H Zhang M An L Wang Y. Effects of different doses lipopolysaccharides on the mucosal barrier in mouse intestine. Res Vet Sci. (2020) 133:75–84. 10.1016/j.rvsc.2020.09.005 32947071

[45] Rooke JA Bland IM. The acquisition of passive immunity in the new-born piglet. Livest Prod Sci. (2002) 78:13–23. 10.1016/S0301-6226(02)00182-3

[46] Guo L Hua J Luan Z Xue P Zhou S Wang X. Effects of the stems and leaves of astragalus membranaceus on growth performance, immunological parameters, antioxidant status, and intestinal bacteria of quail. Anim Sci J. (2019) 90:747–56. 10.1111/asj.13213 30989748

[47] Medzhitov R Janeway C. Innate immunity. N Engl J Med. (2000) 343:338–44. 10.1056/NEJM200008033430506 10922424

[48] Rosadini CV Kagan JC. Early innate immune responses to bacterial LPS. Curr Opin Immunol. (2017) 44:14–9. 10.1016/j.coi.2016.10.005 27842237 PMC5426986

[49] Chen Y Mou D Hu L Zhen J Che L Fang Z. Effects of maternal low-energy diet during gestation on intestinal morphology, disaccharidase activity, and immune response to lipopolysaccharide challenge in pig offspring. Nutrients. (2017) 9:1115. 10.3390/nu9101115 29027951 PMC5691731

[50] Zhu H Wang H Wang S Tu Z Zhang L Wang X. Flaxseed oil attenuates intestinal damage and inflammation by regulating necroptosis and tlr4/nod signaling pathways following lipopolysaccharide challenge in a piglet model. Mol Nutr Food Res. (2018) 62:1700814. 10.1002/mnfr.201700814 29510469

[51] Oeckinghaus A Ghosh S. The NF-kappaB family of transcription factors and its regulation. Cold Spring Harbor Perspect Biol. (2009) 1:a000034. 10.1101/cshperspect.a000034 20066092 PMC2773619

[52] Ali T Rahman SU Hao Q Li W Liu Z Ali Shah F. Melatonin prevents neuroinflammation and relieves depression by attenuating autophagy impairment through FOXO3a regulation. J Pineal Res. (2020) 69:e12667. 10.1111/jpi.12667 32375205

